# Suggestive Therapeutics and Forensic Medicine

**Published:** 1904-11

**Authors:** 


SUGGESTIVE THERAPEUTICS AND FORENSIC MEDICINE are under the management of Dr. D. R. Brown and H. A. Moyer, respectively, and contain in a condensed form the latest contributions to these interesting subjects. Volume for August, 1904.

In this volume the subject of Physiology and Bacteriology is dealt with by A. Gehrmann, of Chicago, Anatomy and Pathology by W. S. Evans, M.S., M.D. The progress in these sciences is brought out in detail. The sections are well illustrated. There is also in the volume an index or dictionary of new words collated by Wm. Heally, A.B., M.D.

